# Laparoscopic Repair of Duodenal Atresia with Concurrent Situs Anomaly: A Case Series and Technical Considerations

**DOI:** 10.3390/children13060749

**Published:** 2026-05-28

**Authors:** Min-Jung Bang, Wontae Kim, Sungjoo Park, Sanghoon Lee, Jeong-Meen Seo

**Affiliations:** 1 Division of Pediatric Surgery, Ajou Medical Center, Ajou University School of Medicine, Suwon 16499, Republic of Korea; mj.bang@aumc.ac.kr; 2 Department of Surgery, CHA Bundang Medical Center, CHA University School of Medicine, Seongnam 13488, Republic of Korea; wnt.kim@chamc.co.kr; 3 Department of Surgery, Samsung Medical Center, Sungkyunkwan University School of Medicine, Seoul 06351, Republic of Korea; sungjoo89.park@samsung.com (S.P.); jm0815.seo@samsung.com (J.-M.S.)

**Keywords:** duodenal atresia, situs anomaly, heterotaxy, laparoscopic surgery, neonate, intestinal malrotation

## Abstract

**Highlights:**

**What are the main findings?**

- Laparoscopic duodenoduodenostomy was successfully completed in all neonates with duodenal atresia and situs anomaly without open conversion, with operative time, feeding milestones, and hospital stay comparable to those with normal situs.
- All situs anomaly cases presented with situs ambiguus, interrupted inferior vena cava with azygos continuation, and intestinal malrotation, yet none experienced anastomotic complications or reoperations.

**What are the implications of the main findings?**

- Complex visceral malposition, including heterotaxy syndrome, should not be considered a contraindication to minimally invasive repair; laparoscopic duodenoduodenostomy is feasible and safe with appropriate preoperative imaging and individualized trocar placement.
- These findings expand the applicability of minimally invasive neonatal surgery to anatomically challenging patients, supporting its adoption in high-volume centers managing duodenal atresia with concurrent laterality defects.

**Abstract:**

**Background:** Laparoscopic duodenoduodenostomy is an established approach for duodenal atresia, yet its applicability in neonates with concurrent situs anomalies remains poorly defined. This study evaluated the feasibility and perioperative outcomes of laparoscopic duodenoduodenostomy in this population. **Methods:** A retrospective review was conducted of all neonates who underwent laparoscopic repair for duodenal atresia by a single surgeon at Samsung Medical Center between January 2017 and December 2023. Thirteen patients were divided into situs anomaly (*n* = 3) and situs solitus (*n* = 10) groups. Anatomical features, operative details, and perioperative outcomes were reviewed and descriptively summarized according to situs status. **Results:** All three neonates in the situs anomaly group had situs ambiguus—left isomerism with polysplenia (*n* = 2) or right isomerism with asplenia (*n* = 1). Interrupted inferior vena cava with azygos continuation and intestinal malrotation were present in all three patients; however, only two required a concurrent Ladd procedure. Laparoscopic repair was completed in all 13 patients without open conversion. Operative time, feeding milestones, and hospital stay were descriptively similar between groups. No anastomotic complications, reoperations, or mortality occurred. **Conclusion:** In this small case series, laparoscopic duodenoduodenostomy was completed without conversion or perioperative anastomotic complications in neonates with duodenal atresia and concurrent situs anomalies. Situs anomaly alone may not preclude minimally invasive repair in experienced hands.

## 1. Introduction

Duodenal atresia, affecting approximately 1 in 5000–10,000 live births, results from failed duodenal recanalization during embryogenesis and frequently presents with concomitant anomalies including Down syndrome, malrotation, and situs anomalies [1,2]. Situs anomalies—encompassing situs inversus and heterotaxy syndrome—occur in approximately 1 in 10,000 births and arise from disrupted left–right axis determination. When concurrent with duodenal atresia, these laterality defects introduce significant surgical complexity due to altered anatomical landmarks, variant vascular patterns, and unexpected organ positioning.

Since Rothenberg’s initial description in 2002, laparoscopic duodenoduodenostomy has gained wide acceptance as an alternative to open repair, offering reduced postoperative pain, earlier feeding, and shorter hospitalization [3,4,5,6]. However, existing studies have systematically excluded patients with situs anomalies, leaving a critical knowledge gap regarding the applicability of minimally invasive repair in this population [7,8,9,10]. Concerns about spatial disorientation and the need for technical adaptation have traditionally led surgeons to prefer open repair in such cases.

Importantly, laparoscopic duodenoduodenostomy employs a symmetric anastomotic configuration that remains structurally consistent regardless of visceral laterality, as the duodenum occupies a central retroperitoneal position largely unaffected by situs. This inherent symmetry suggests that the laparoscopic approach may, in fact, be particularly well-suited for patients with situs anomalies, with technical modifications primarily required for any concurrent Ladd procedure rather than for the anastomosis itself. We present a case series of three neonates with duodenal atresia and concurrent situs anomaly who underwent laparoscopic repair, with the aim of describing operative findings, technical adaptations, and short-term outcomes to inform surgical decision-making in this rare but anatomically complex population.

## 2. Materials and Methods

### 2.1. Study Design and Patient Selection

This retrospective review included all neonates who underwent laparoscopic repair for congenital duodenal obstruction between June 2017 and December 2023 at Samsung Medical Center. Inclusion criteria were (1) confirmed duodenal atresia or stenosis, (2) presence of situs anomaly verified by imaging or intraoperative findings, and (3) laparoscopic approach as the primary technique. During the study period, no patients with duodenal atresia and situs anomaly underwent primary open repair at our institution.

### 2.2. Definition and Evaluation of Situs Anomalies

Situs inversus was defined as a complete mirror-image reversal of normal thoracoabdominal organ arrangement. Heterotaxy syndrome (situs ambiguus) was defined as abnormal organ arrangement along the left–right axis without complete reversal, and further classified into right isomerism (asplenia) and left isomerism (polysplenia) based on splenic status, hepatic position, and cardiac morphology. Situs anomalies were diagnosed using multimodal imaging, including prenatal ultrasonography or MRI when available, and postnatal chest radiography, echocardiography, abdominal ultrasonography, or contrast studies. Duodenal obstruction was confirmed by radiographic “double-bubble” findings. Associated anomalies such as cardiac defects, malrotation, and biliary anomalies were also assessed.

### 2.3. Data Collection and Outcomes

Medical records and operative videos were retrospectively reviewed for demographic, clinical, radiologic, and operative data, as well as postoperative outcomes. Primary outcomes were technical feasibility (completion without conversion) and 30-day morbidity; secondary outcomes included operative time, feeding milestones, hospital stay, and complications graded by the Clavien–Dindo system.

### 2.4. Surgical Technique

After general anesthesia, all patients were placed in the supine position. A 5-mm umbilical port was inserted for a 30° laparoscope, with two additional 3-mm working ports placed in the right and left lower quadrants. Notably, trocar placement was maintained in the standard configuration across all cases, including those with situs anomaly, as the duodenum occupies a central retroperitoneal position that is largely unaffected by visceral laterality. Liver retraction was achieved using a transabdominal stay suture in all cases.

Upon entering the abdomen, the rotation status of the intestine was assessed prior to duodenal repair. In Cases 1 and 2, intestinal malrotation was identified, and a Ladd procedure was performed prior to duodenoduodenostomy. Critically, the bowel arrangement following the Ladd procedure was executed in a completely mirror-image fashion compared to the standard technique, with the colon positioned on the right and the small bowel on the left, in accordance with the patient’s situs anomaly. Mesenteric widening was performed as part of the Ladd procedure in these cases. In Case 3, non-rotation of the intestine was confirmed intraoperatively; as the mesentery was already broadly based without pathological fixation, no Ladd procedure or mesenteric widening was required, and laparoscopic duodenoduodenostomy was performed without additional technical modification.

Following assessment of intestinal rotation, the proximal dilated and distal collapsed duodenum were identified and mobilized. A side-to-side parallel duodenoduodenostomy was constructed using interrupted 4-0 absorbable sutures. The anastomotic configuration remained structurally consistent regardless of visceral laterality, as the symmetric nature of the parallel anastomosis requires no fundamental modification in the setting of situs anomaly [11]. This inherent symmetry of laparoscopic duodenoduodenostomy may represent a technical advantage over open repair in patients with altered visceral orientation.

### 2.5. Statistical Analysis

Demographic, operative, and postoperative data were analyzed descriptively. Given the exploratory nature of this case series and the small number of patients with situs anomaly, no inferential statistical comparisons were performed. Continuous variables are expressed as means ± standard deviation or medians with ranges, and categorical variables as counts and percentages. Statistical analyses were performed using SPSS version 29.0 (IBM Corp., Armonk, NY, USA).

## 3. Results

### 3.1. Patient Characteristics

During the study period, a total of 13 neonates underwent laparoscopic repair for congenital duodenal obstruction by a single surgeon. Of these, 10 patients (76.9%) had situs solitus, and 3 (23.1%) had situs anomalies. No patients were excluded or converted to open surgery during this period. Table 1 summarizes the clinical characteristics and prenatal findings of the three neonates with situs anomaly. The median gestational age was 37 + 5 weeks, and the mean birth weight was 2805 g. All three underwent laparoscopic duodenoduodenostomy without intraoperative complications.

### 3.2. Associated Anomalies

Visceral configurations are summarized in Table 2. All three patients had a transversely located liver and a right-sided stomach. Two had polysplenia (left isomerism), and one had asplenia (right isomerism). Interruption of the inferior vena cava with azygos continuation was present in all patients. Malrotation was present in all three patients (100%). Cases 1 and 2 had malrotation with pathological fixation requiring a concurrent Ladd procedure, whereas Case 3 had non-rotation—a subtype of malrotation—with a broadly based mesentery and no pathological fixation, for which no Ladd procedure or mesenteric widening was deemed necessary. Biliary atresia was absent in all cases.

Cardiovascular anatomy is described in Table 3. Cases 1 and 3 had levocardia, and Case 2 had mirror-image dextrocardia. The aortic arch was left-sided in two patients and right-sided in one. Systemic venous drainage patterns varied among cases, including single right SVC, single left SVC, and bilateral SVCs with azygos continuation. All three patients had associated congenital heart disease: two had complex cardiac malformations (atrioventricular septal defect and double-outlet right ventricle), and one had a mild anomaly not requiring surgical intervention.

### 3.3. Case-Specific Operative Findings

**Figure 1:**
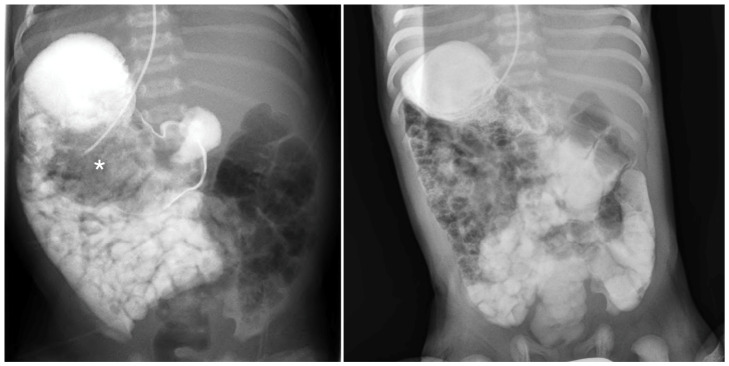
Upper gastrointestinal series of a male neonate with situs anomaly demonstrating a right-sided stomach, marked by an asterisk (*), with delayed passage of contrast through a duodenal web on the fourth day of life.

In Case 1, preoperative imaging suggested a situs anomaly with a right-sided stomach, transverse liver, interrupted inferior vena cava with azygos continuation, and intestinal malrotation. During laparoscopy, malrotation with abnormal peritoneal bands and a narrow mesenteric base was confirmed. A Ladd procedure was performed in a mirror-image fashion, with division of obstructing bands and broadening of the mesenteric base, followed by parallel duodenoduodenostomy. The duodenal anastomosis itself required no fundamental modification, although careful orientation of the proximal dilated and distal collapsed duodenal segments was necessary (Figure 1). 

**Figure 2:**
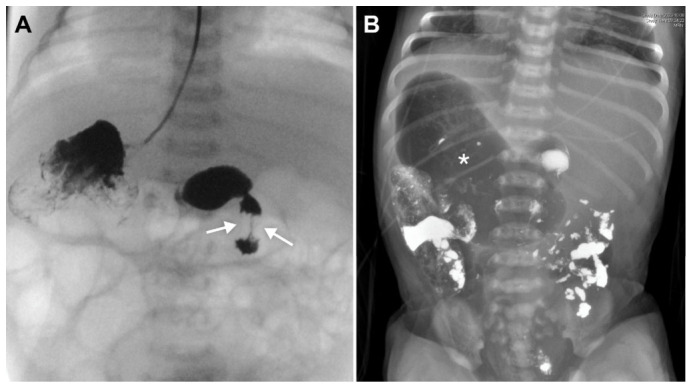
(**A**) Upper gastrointestinal series of a female neonate with situs anomaly demonstrating the site of duodenal obstruction, indicated by a white arrow, on the fourth day of life. (**B**) Post-upper gastrointestinal series abdominal radiograph of the same patient showing a mirror-image distended stomach, marked by an asterisk (*), and left-sided duodenum on the third day of life.

In Case 2, prenatal suspicion of situs inversus was revised postnatally to situs ambiguus based on comprehensive imaging evaluation. Laparoscopy confirmed heterotaxy with a right-sided stomach and malrotation. Similar to Case 1, the Ladd procedure was performed with a mirror-image bowel arrangement, positioning the colon on the right and the small bowel on the left. After correction of the rotational abnormality, the duodenoduodenostomy was completed using the standard parallel anastomotic technique (Figure 2). 

**Figure 3:**
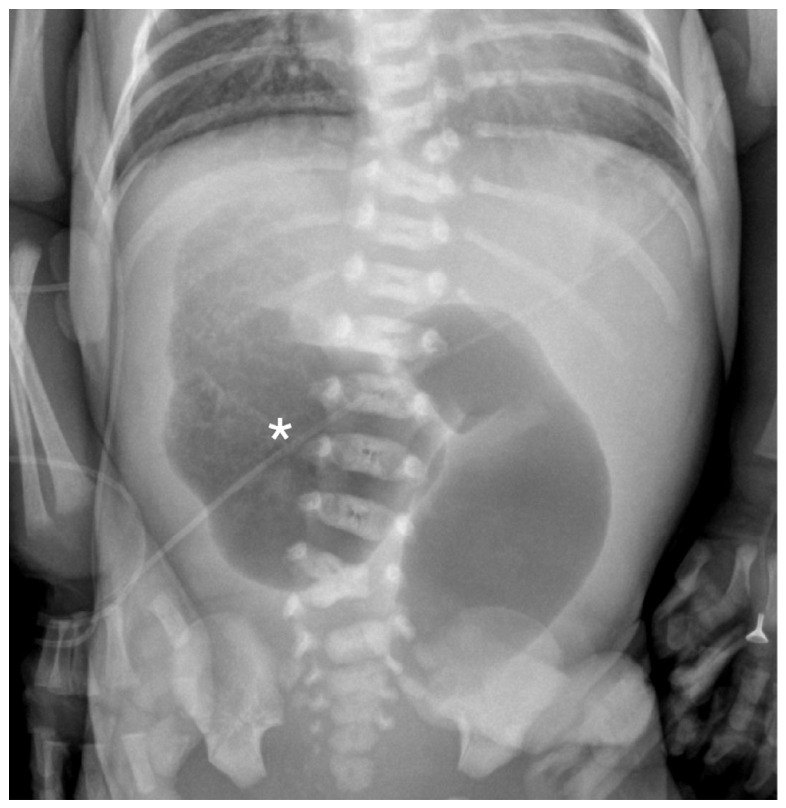
Abdominal radiograph of a female neonate with situs anomaly demonstrating a reversed double-bubble sign on the second day of life. The asterisk (*) indicates the right-sided stomach.

In Case 3, preoperative evaluation demonstrated duodenal obstruction with situs anomaly, and intraoperative assessment revealed non-rotation without pathological fixation or a narrow mesenteric pedicle. Because the mesentery was already broadly based, no Ladd procedure or additional mesenteric widening was required. Laparoscopic duodenoduodenostomy was therefore performed without major technical modification (Figure 3). These case-specific findings suggest that the operative complexity in patients with situs anomaly is influenced primarily by the associated rotational anatomy rather than by the duodenal anastomosis itself.

### 3.4. Clinical Outcomes

Perioperative outcomes are summarized in Table 4. Given the small sample size, particularly in the situs anomaly group, no inferential statistical comparisons were performed. The median gestational age (36 + 6 vs. 37 + 5 weeks) and birth weight (2637 g vs. 2853 g) were similar between the situs solitus and situs anomaly groups. Operative time was comparable between groups (156.9 vs. 163.0 min). Time to first oral feeding was 6.5 ± 3.4 days in the situs solitus group and 7.0 ± 2.6 days in the situs anomaly group. Time to full enteral feeding was 12.9 ± 5.6 and 18.3 ± 8.2 days, respectively. Hospital stay tended to be longer in the situs anomaly group (34.0 ± 9.6 vs. 23.9 ± 12.3 days). Malrotation was present in all three patients with situs anomaly (100%) and in two of ten patients with situs solitus (20%). No patient in either group experienced anastomotic leakage, postoperative stenosis, or mortality. The median follow-up duration was 18.5 months (range, 3–39 months) in the situs solitus group and 4 months (range, 2–4 months) in the situs anomaly group. All three patients with situs anomaly remained clinically well at the latest follow-up, with no clinically apparent delayed procedure-related complications.

## 4. Discussion

Duodenal atresia is rarely associated with a situs anomaly. In a 25-year Korean single-center review, only 1 out of 26 neonates (3.8%) with situs anomalies had duodenal atresia [1]. Similarly, a population-based U.S. study identified duodenal atresia in only 2 of 517 neonates (0.4%) with laterality defects, underscoring the rarity of this association [2,12,13].

This study evaluated the feasibility of laparoscopic duodenoduodenostomy in neonates with duodenal atresia and concurrent situs anomalies. All procedures were completed laparoscopically without conversion to open surgery, and no major intraoperative complications occurred. Operative times, postoperative feeding progression, and lengths of hospitalization were similar to those of the situs solitus cohort, suggesting that complex visceral malposition may not preclude successful minimally invasive repair in experienced hands.

A key technical implication of this case series is that the presence of situs anomaly alone may not justify excluding neonates with duodenal atresia from a laparoscopic approach, provided that associated intestinal rotational abnormalities are carefully recognized and managed. In our experience, intraoperative complexity was driven primarily by the need for mirror-image bowel arrangement during the Ladd procedure rather than by modification of the duodenoduodenostomy itself.

In Cases 1 and 2, a concurrent Ladd procedure was required, with bowel arrangement executed in a completely mirror-image fashion compared to the standard technique—placing the colon on the right and the small bowel on the left. This represents the most critical technical adaptation in the setting of a situs anomaly. In contrast, Case 3, who had non-rotation without pathological fixation, underwent straightforward laparoscopic duodenoduodenostomy without mesenteric widening or bowel repositioning, demonstrating that situs anomaly alone does not necessarily add significant technical complexity. Importantly, the anastomotic configuration itself required no fundamental modification across all cases, as the symmetric nature of parallel laparoscopic duodenoduodenostomy remains structurally consistent regardless of visceral laterality. Trocar placement was also maintained in the standard position in all cases, owing to the central retroperitoneal location of the duodenum.

The scarcity of systematic evidence regarding laparoscopic repair in this population has traditionally led to a preference for open surgery [14,15]. Our findings suggest that laparoscopy may be a viable option in experienced hands, with technical modifications primarily required for any concurrent Ladd procedure rather than for the anastomosis itself. However, given the small sample size and single-center experience, these observations should be regarded as preliminary and hypothesis-generating rather than practice-changing.

Prior literature on this topic has been limited to isolated case reports. Habib et al. described successful laparoscopic repair in a neonate with situs inversus totalis [16], and Shcherbinin et al. demonstrated feasibility in both situs inversus and heterotaxy variants [17]. Our series adds to these preliminary observations, suggesting that laparoscopic repair may be feasible across the spectrum of situs anomalies when performed at experienced centers with appropriate preoperative planning.

Several limitations of this study must be acknowledged. The small sample size, particularly in the situs anomaly group (*n* = 3), significantly limits statistical power and generalizability, and all comparative data should be interpreted as descriptive only. The retrospective design and single-surgeon, single-center experience further limit broader applicability. Additionally, although no unplanned hospital visits or readmissions related to duodenal repair were documented in the situs anomaly group, the absence of scheduled long-term outpatient follow-up and the short follow-up duration limit definitive assessment of late complications, such as adhesive bowel obstruction, growth-related sequelae, or late anastomotic problems. The difference in follow-up duration between groups should also be interpreted cautiously. The longer follow-up in the situs solitus group may partly reflect the need for ongoing surveillance of associated comorbidities, including imperforate anus and tracheoesophageal fistula, whereas the situs anomaly group had only short-term postoperative follow-up of 2–4 months. Future multicenter prospective studies are needed to validate these preliminary findings.

## 5. Conclusions

In this small case series, laparoscopic duodenoduodenostomy was completed without conversion or perioperative anastomotic complications in three neonates with duodenal atresia and concurrent situs anomalies. The primary technical adaptation required was a mirror-image bowel arrangement during the concurrent Ladd procedure, while the anastomotic technique itself required minimal modification owing to the inherent symmetry of the parallel anastomotic configuration. These preliminary findings suggest that situs anomaly alone may not preclude minimally invasive repair in experienced hands, though larger multicenter studies are needed to confirm these observations.

## Figures and Tables

**Table 1:** Clinical Presentation and Prenatal Diagnosis of Three Neonates with Duodenal Atresia and Situs Anomaly.

Case	Sex	Gestational Age (Weeks)	Birth Weight (g)	Prenatal Diagnosis		Presenting Symptom	Type
				Heart	Abdomen		
1	M	39 + 6	3410	situs inversus *	-	Bilious vomiting	Duodenal web
2	F	39 + 5	3220	situs inversus *	-	Bilious vomiting	Duodenal web
3	F	33 + 5	1930	-	Duodenal atresia	-	Duodenal web

* Prenatal ultrasonographic impression of situs inversus was revised to situs ambiguus (heterotaxy syndrome) following comprehensive postnatal evaluation, including echocardiography.

**Table 2:** Abdominal Organ Positioning and Associated Anomalies.

Case	Abdominal Situs	Liver	Stomach	Spleen	Interrupted IVC	Malrotation	Biliary Atresia
1	Ambiguus	Transverse	Rt. side	Asplenia	Yes	Yes	No
2	Ambiguus	Transverse	Rt. side	Polysplenia	Yes	Yes	No
3	Ambiguus	Transverse	Rt. side	Polysplenia	Yes	Yes	No

IVC, inferior vena cava.

**Table 3:** Cardiovascular Anomalies and Venous Drainage Patterns.

Case	Heart	Aortic Arch	SVC	IVC Drainage	
				Interruption	Continuation
1	Levocardia	Lt. side	Single Rt side	azygos	Rt. SVC
2	Mirror-image dextrocardia	Rt. side	Single Lt side	azygos	Lt. SVC
3	Levocardia	Lt. side	Bilateral	azygos	Rt. SVC

IVC, inferior vena cava; SVC, superior vena cava.

**Table 4:** Summary of Surgical and Postoperative Outcomes According to Situs Status.

	Situs Solitus (*N* = 10)	Situs Anomaly (*N* = 3)
Gestational age (wks)	36 + 6	37 + 5
Birth weight (g)	2637	2853.3
Malrotation (%)	2 (20)	3 (100)
Operative time (min)	156.9	163
Time to first oral feeding (days)	6.5	7
Time to full enteral feeding (days)	12.9	18.3
Length of hospital stay (days)	23.9	34
Anastomotic leakage	0	0
Anastomotic stenosis	0	0
Follow-up duration (months)	18.5 (3–39)	4 (2–4)

Values are presented as mean, median (range), or number (%), as appropriate.

## Data Availability

The data presented in this study are available on request from the corresponding author. The data are not publicly available due to privacy restrictions related to patient medical records.
